# Histopathological comparison of Sjögren-related features between paired labial and parotid salivary gland biopsies of sicca patients

**DOI:** 10.1093/rheumatology/keae154

**Published:** 2024-03-27

**Authors:** Uzma Nakshbandi, Martha S van Ginkel, Gwenny M P J Verstappen, Fred K L Spijkervet, Suzanne Arends, Erlin A Haacke, Silvia C Liefers, Arjan Vissink, Hendrika Bootsma, Frans G M Kroese, Bert van der Vegt

**Affiliations:** Department of Rheumatology and Clinical Immunology, University of Groningen and University Medical Center Groningen, Groningen, The Netherlands; Department of Rheumatology and Clinical Immunology, University of Groningen and University Medical Center Groningen, Groningen, The Netherlands; Department of Rheumatology and Clinical Immunology, University of Groningen and University Medical Center Groningen, Groningen, The Netherlands; Department of Oral and Maxillofacial Surgery, University of Groningen and University Medical Center Groningen, Groningen, The Netherlands; Department of Rheumatology and Clinical Immunology, University of Groningen and University Medical Center Groningen, Groningen, The Netherlands; Department of Pathology, Pathology Friesland, Leeuwarden, The Netherlands; Department of Rheumatology and Clinical Immunology, University of Groningen and University Medical Center Groningen, Groningen, The Netherlands; Department of Oral and Maxillofacial Surgery, University of Groningen and University Medical Center Groningen, Groningen, The Netherlands; Department of Rheumatology and Clinical Immunology, University of Groningen and University Medical Center Groningen, Groningen, The Netherlands; Department of Rheumatology and Clinical Immunology, University of Groningen and University Medical Center Groningen, Groningen, The Netherlands; Department of Pathology and Medical Biology, University of Groningen and University Medical Center Groningen, Groningen, The Netherlands

**Keywords:** Sjögren’s disease, histopathology, gland biopsy, sicca, labial gland, parotid salivary gland

## Abstract

**Objectives:**

To compare focus score and other histopathological features between paired labial and parotid salivary gland biopsies in a diagnostic cohort of suspected Sjögren’s disease (SjD) patients.

**Methods:**

Labial and parotid salivary gland biopsies were simultaneously obtained from patients with sicca complaints, suspected of having SjD. Biopsies were formalin fixed and paraffin embedded. Sections were stained with haematoxylin & eosin, and for CD3, CD20, CD45, cytokeratin, CD21, Bcl6, activation-induced deaminase (AID) and IgA/IgG. Focus score and other histopathological features characteristic for SjD were analysed.

**Results:**

Based on the expert opinion of three experienced rheumatologists, 36 patients were diagnosed as SjD and 63 as non-SjD sicca patients. When taking all patients together, absolute agreement of various histopathological features between labial and parotid biopsies was high and varied between 80% (focus score) and 93% [(pre-)lymphoepithelial lesions (LELs)]. More labial gland biopsies had a focus score ≥1 compared with their parotid counterpart. Accordingly, the area of infiltrate was larger in labial gland biopsies. When considering only SjD patients, labial glands contained significantly fewer B-lymphocytes and germinal centres/mm^2^, and less severe LELs compared with parotid glands.

**Conclusion:**

Labial and parotid glands from SjD patients contain similar histopathological key features, and thus both glands can be used for diagnosis and classification of SjD. However, parotid salivary glands reveal more evident B-lymphocyte-related features, while labial glands exhibit more inflammation, which may be partially unrelated to SjD.

Rheumatology key messages

- Histopathological key-features characteristic for Sjögren’s disease (SjD) were observed at a similar frequency in both labial and parotid glands from SjD patients.
- Parotid glands reveal more evident histopathological signs of B-cell hyperactivity compared with labial glands.
- Labial gland biopsies showed more inflammation, however this might not be completely related to SjD.

## Introduction

Sjögren’s disease (SjD) is a systemic autoimmune disease, characterized by chronic inflammation of salivary and lacrimal glands. Patients typically present with dryness complaints such as xerostomia and keratoconjunctivitis sicca [1]. In salivary glands of SjD patients, the chronic inflammation is a focal lymphocytic sialadenitis characterized by lymphocytic foci commonly associated with striated ducts.

Traditionally, a labial salivary gland biopsy is obtained for diagnosis and classification. A parotid biopsy has comparable sensitivity and specificity to a labial biopsy, making it a good alternative [2]. The patient-reported postoperative changes in sensibility and pain in the area of the parotid and labial gland biopsy are minor and comparable [3]. The applicability of the parotid biopsy has increased since the introduction of US-guided core needle biopsies, showing comparable results to incisional parotid gland biopsies [4]. Moreover, parotid gland biopsies have several advantages including the possibility to perform a repeated biopsy from the same gland and the higher likelihood of detecting a mucosa-associated lymphoid tissue (MALT) lymphoma compared with labial gland biopsies.

Salivary gland biopsies have a prominent position in the ACR-EULAR classification criteria for SjD [5]. In these criteria, histopathological classification of SjD is only based on the focus score [6, 7]. A focus is defined as a cluster of ≥50 lymphocytes and the focus score is the number of foci per 4-mm^2^ salivary gland tissue. In both labial and parotid gland biopsies, a focus score ≥1 is considered positive for SjD. While focus score is used as a classification tool, it is only based on the number of foci and does not consider the size of the inflammatory infiltrates. Therefore, others have proposed to use the total size of these focal infiltrates as an alternative for the focus score, thereby providing a better quantification of the extent of glandular inflammation [8, 9].

Although lymphocytic foci are characteristic for SjD, they are certainly not the only histopathological key feature of the inflammatory infiltrate. Lymphocytic foci potentially evolve under influence of chemokines and cytokines into ectopic lymphoid structures that exhibit segregated T- and B-cell areas with follicular dendritic cell (FDC) networks and possibly even germinal centres (GCs) [10, 11]. GCs typically express transcription factor Bcl-6 and the enzyme activation-induced cytidine deaminase (AID), the enzyme responsible for somatic hypermutation and class switch recombination in the IgG genes of B-lymphocytes [12]. Other characteristic histopathological features found in salivary glands of SjD patients comprise a relative increase in the number of IgG-producing plasma cells with a concomitant relative decrease in the number of IgA plasma cells (the so-called plasma cell immunoglobulin isotype shift), and the presence of lymphoepithelial lesions (LELs) [13, 14]. LELs are defined as striated ducts infiltrated by B lymphocytes with concurrent hyperplasia of the ductal epithelium [15]. B lymphocytes infiltrating the ductal epithelium may precede hyperplasia of the epithelium and ducts with intraepithelial B lymphocytes, but without hyperplasia, are therefore called pre-LELs [16]. Presence of GCs, plasma-cell shift and LELs reflect the hallmark finding of B-lymphocyte hyperactivity in SjD [10, 17]. Recently, we have demonstrated that addition of two of these histopathological features to the focus score increases diagnostic accuracy of the labial gland biopsy for SjD [18].

While all these histopathological features can be seen in both minor (labial) and major (parotid) salivary glands, it is unclear whether all these features develop simultaneously in both gland types in an individual patient. This might be relevant for classification, diagnosis and prognosis, and also may increase our understanding of the pathogenesis of the disease. Therefore, the aim of this study was to compare focus score and other histopathological features between paired labial and parotid salivary gland biopsies of SjD and non-SjD sicca patients.

## Methods

### Patients

In this prospective study, consecutive patients with oral and/or ocular sicca complaints, suspected of having SjD, who underwent a full diagnostic workup at the University Medical Center Groningen (UMCG), a tertiary referral centre and centre of expertise for SjD, were included between 2014 and 2017. Labial and parotid gland biopsies were simultaneously obtained under local infiltration anaesthesia by the same oral and maxillofacial surgeon (F.K.L.S.) [19]. Exclusion criteria for this study were: presence of another associated autoimmune disease, positive hepatitis C serology, salivary gland MALT lymphoma, sclerosing sialadenitis and insufficient biopsy material (total surface area of sections <1 mm^2^). Participants gave written consent according to the declaration of Helsinki. The study was approved by the Medical Research Ethics Committee of the UMCG, the Netherlands (METc2013.066).

### Clinical evaluation

All patients were diagnosed as SjD or non-SjD sicca based on the expert opinion of three experienced rheumatologists (H.Bootsma, A.J. Stel, L. Brouwer). The expert panel had access to anonymized clinical vignettes including all signs and symptoms, medication use, lab tests clinical parameters and focus score of labial and parotid gland biopsies. Disagreement was resolved during a consensus meeting (for details see van Ginkel *et al.* in press) [18].

### Histochemical and immunohistochemical staining

Biopsy material was formalin fixed (4%), paraffin embedded and serially sectioned at 3-µm thickness. After deparaffinization, sections were stained for haematoxylin & eosin (H&E) or by immunohistochemistry. Immunohistochemical staining was performed either manually or using an automated staining platform (Benchmark XT, Ventana Medical Systems, Inc. Oro Valley, Arizona, USA) (see Supplementary Table S1, available at *Rheumatology* online). For manual immunohistochemical staining antigen retrieval was carried out by incubating the tissue sections for 15 min with EDTA buffer, pH 8.0. Endogenous peroxidase activity was blocked using H_2_O_2_. Hereafter, slides were incubated with primary antibodies for 75 min and a poly-horseradish peroxidase (HRP)-labelled secondary antibody (Thermo Fisher Scientific). AID staining was performed as follows: after deparaffinization, antigen retrieval was performed overnight using Tris–HCl buffer with a pH of 9.0 at 80°C. Endogenous peroxidase activity was blocked using H_2_O_2_. Hereafter, slides were incubated with primary antibody, rat anti-human AID, for 30 min. After incubation with a HRP-labelled rabbit anti-rat IgG secondary antibody (Invitrogen), an HRP-labelled goat anti-rabbit IgG tertiary antibody (Dako) and HRP-labelled rabbit anti-goat IgG quaternary antibody (Dako). Antibodies for all manual stainings were visualized by using DAB (3,3′ diaminobenzidine) and slides were counterstained with haematoxylin. Automated staining was performed according to the manufacturer’s protocols. All stained slides were digitized using a Philips UFS slide scanner (Philips, Best, The Netherlands) and assessed using Philips IntelliSite Pathology Solution software. The focus score was calculated on a whole H&E-stained salivary gland section (B.v.d.V., E.A.H.). Discrepancies were resolved during a consensus meeting.

Quantitative digital image analyses of salivary gland sections stained for CD3^+^ T-lymphocytes, CD20^+^ B-lymphocytes and CD45^+^ lymphocytic infiltrates were performed using QuPath v0.1.2 [20]. For each section, the total area of parenchyma was evaluated by defining regions of interest using the Simple Tissue Detection application (threshold 215), excluding extra- and intra-parenchymal areas with adipose tissue. Hereafter, atrophic and extra-parenchymal fibrotic areas were manually excluded. All algorithms were verified by an experienced head and neck pathologist (B.v.d.V.).

### Histopathological analyses

To assess the area of the parenchyma that was infiltrated by CD45^+^ lymphocytic infiltrates, the so-called ‘cytokeratin annotation’ function in QuPath was used to select DAB positive areas. The threshold between CD45 positive and negative areas was set to 0.15. The area of CD45^+^ infiltrate was calculated as a percentage of the area of parenchymal tissue (Supplementary Fig. S1A and B, available at *Rheumatology* online).

The Positive Cell Detection algorithm was used to select DAB-positive cells and an Object Classifier was used to adjust the algorithm. Hereafter, the number of CD3 and CD20 DAB-positive cells was calculated per mm^2^ of parenchymal tissue (Supplementary Fig. S1C and D, available at *Rheumatology* online).

FDC networks were identified by positive CD21 staining, and the number of FDC networks per mm^2^ parenchymal tissue was manually counted. GCs were identified by positive Bcl6 staining. GCs were defined as a cluster of five or more Bcl6-positive cells [11]. The number of GCs/mm^2^ was manually assessed. All sections with a CD21^+^ FDC network were stained for AID as an alternative functional marker for GCs. GCs were defined as a cluster of five or more AID-positive cells. As a negative control, 10 sections with foci but without a CD21^+^ FDC network were also stained.

In order to estimate the percentages of IgA^+^ and IgG^+^ plasma cells, biopsies were double stained for IgA and IgG, and manually evaluated. A percentage of >30% IgG^+^ plasma cells of all IgA and IgG plasma cells was considered as a threshold for an IgA/IgG shift [13].

The grade of organization of the lymphocytic infiltrate was assessed for all individual foci and the highest grade was noted per section. Grade of organization was defined as follows (Supplementary Fig. S2, available at *Rheumatology* online). Grade 1: lymphocytic foci were present, but a clear T/B-lymphocyte segregation was lacking based on the CD3 and CD20 stainings, and FDC networks and GCs were absent. Grade 2: either T/B-cell segregation within a focus and/or an FDC network was present, but without presence of a GC. Grade 3: grade 2 features accompanied by the presence of a GC.

For the assessment of pre-LELs and LELs, high molecular-weight cytokeratin and CD20 stainings were performed on consecutive sections. After alignment of sections, a digital image analyses algorithm in Visiopharm Integrator System (Hørsholm, Denmark) was used to identify pre-LELs and LELs, as previously described [16]. Presence of intraepithelial CD20^+^ B lymphocytes was assessed manually when high molecular-weight cytokeratin and CD20-stained sections could not be aligned. The number of (pre-)LEL/mm^2^ and the maximum severity of LELs was scored as previously described [16] with the addition of pre-LELs. LEL stages were defined as follows. Stage 0 LEL (i.e. pre-LEL): presence of intraepithelial B lymphocytes without ductal hyperplasia. Stage 1 LEL: lymphocytic ductal infiltration and ductal hyperplasia affecting <50% of the epithelium. Stage 2 LEL: lymphocytic ductal infiltration and ductal hyperplasia affecting between 50% and 100% of the epithelium. Stage 3 LEL: lymphocytic ductal infiltration and fully circumferentially hyperplastic epithelium without lumen.

Sections were analysed by trained researchers (U.N., M.S.v.G., S.C.L., E.A.H.) under supervision of an experienced head and neck pathologist (B.v.d.V.). Disagreements were resolved during a consensus meeting.

### Statistical analysis

Data were analysed using SPSS version 28 statistical software (SPSS Inc., Chicago, IL, USA). Results were expressed as number of patients (%), mean ± s.d. or median (IQR) for categorical, normally distributed and non-normally distributed data, respectively. Differences in clinical and histopathological parameters between SjD and non-SjD sicca patients were tested with χ^2^ or Fisher’s exact test, independent samples *t*-test and Mann–Whitney U test when appropriate. Histopathological features of paired parotid and labial salivary gland biopsies were compared with McNemar’s test and Wilcoxon signed-rank test, and by calculating the absolute agreement. The association between histopathological features in the parotid and labial glands were analysed using Spearman correlation coefficient (ρ), and interpreted as poor (0.0–0.2), fair (0.2–0.4), moderate (0.4–0.6), good (0.6–0.8) or excellent (0.8–1.0). *P*-values <0.05 were considered statistically significant.

## Results

### Patients

From a diagnostic cohort, 99 out of 111 consecutive patients with sicca complaints were included in the analyses. Patients were excluded from this study due to presence of another associated autoimmune disease (*n* = 7), hepatitis C infection (*n* = 1), parotid MALT lymphoma (*n* = 2), sclerosing sialadenitis (*n* = 1) or insufficient biopsy material (*n* = 1). Of the 99 included patients, 36 patients were categorized as SjD and 63 patients as non-SjD sicca by the expert panel. Demographic, serological and clinical characteristics of SjD patients and non-SjD sicca patients are shown in Table 1. As expected, clinical and serological disease characteristics were more frequently present in SjD patients compared with non-SjD sicca patients.

**Table 1.** Clinical and serological parameters of SjD patients and non-SjD sicca patients

	SjD patients (*n* = 36)	Non-SjD sicca patients (*n* = 63)	*P*-value
Clinical parameters			
Age, years	51 ± 14	50 ± 13	0.38
Female, (%)	35 (97.2)	54 (85.6)	0.09
Caucasian, *n* (%)	33 (91.7)	59 (93.7)	0.33
ACR-EULAR+	34 (94.4)	9 (14.3)	<0.001
ESSDAI score	4 (2–12)	1 (0–3)	<0.001
ESSDAI glandular domain	0 (0–2)	0 (0–0)	0.001
Schirmer ≤5 mm, *n* (%)	36 (57.1)	29 (80.6)	0.032
OSS ≥5, *n* (%)	15 (44.1)	8 (12.7)	<0.001
UWS <0.10 ml/min, *n* (%)	20 (55.6)	24 (38.1)	0.10
Serological parameters			
Anti-SSA positive, *n* (%)	29 (80.6)	6 (9.5)	<0.001
Anti-SSB positive, *n* (%)	16 (44.4)	0 0	<0.001
RF positive, *n* (%)	25 (69.4)	3 (4.8)	<0.001
IgG (g/l)	17.1 (12.6–20.0)	10.4 (8.7–12.3)	<0.001
ESR (mm/h)	25.0 (15.0–46.5)	9.5 (4.0–17.0)	<0.001
CRP (mg/l)	2.5 (1.0–5.0)	1.1 (0.5–4.0)	0.06

Data are represented as mean ± SD, median (interquartile range) or *n* (%). Underlined values indicate *P*-values <0.05.

SjD: Sjögren’s disease; ESSDAI: European League Against Rheumatism SS Disease Activity Index; OSS: Oscular Staining Score; UWS: unstimulated whole saliva.

Among the patients diagnosed with SjD by the experts, nearly all patients were also classified as SjD, according to the ACR-EULAR criteria. However, two of these patients did not meet the ACR-EULAR classification criteria. Both patients presented with a positive parotid gland biopsy, and one out of two also had a positive labial gland biopsy (see Supplementary Table S2, available at *Rheumatology* online). The rationale for SjD diagnosis by the expert for patient number 1, in addition to a positive parotid gland biopsy, was based on high disease activity reflected by an ESSDAI score of 18. For patient number 2, indications for SjD included a positive family history and the presence of RP.

Among the non-SjD sicca patients, nine individuals did meet the ACR-EULAR classification criteria. The total ACR-EULAR points of these patients ranged from 4 to 6. Five out of these nine patients exhibited a focus score ≥1 in their labial gland biopsy along with one of the minor criteria (Schirmer’s test ≤5 mm/min or UWS ≤0.1 ml/min) but without a positive parotid gland biopsy or the presence of anti-SSA autoantibodies. One patient had both a positive labial gland and parotid gland biopsy and officially tested positive for anti-SSA autoantibodies. Despite meeting the classification criteria, experts opted not to diagnose SjD as the SSA titre was only 17, and sicca complaints were attributed to comorbidities such as diabetes mellitus type 2. Three non-SjD sicca patients fulfilled the classification criteria based on anti-SSA positivity in combination with minor items but lacked a positive labial or parotid gland biopsy.

### Comparison of histopathological features between paired labial and parotid gland biopsies

#### Total group of sicca patients suspected of having SjD

First, we performed a pairwise analysis of all labial and parotid biopsies from SjD patients and non-SjD sicca patients together. Absolute agreement between labial and parotid glands was high, being 80% for the focus score, 89% for GCs, 84% for the IgA/IgG plasma-cell shift and 93% for (pre-)LELs (see Table 2). In the total study population, a focus score ≥1 was more often observed in labial glands compared with parotid glands (*P* = 0.012). The presence of other histopathological key features, namely presence of GCs, IgA/IgG plasma-cell shift and (pre-)LELs, did not differ significantly between the paired biopsies (Table 2). The higher number of labial gland biopsies with a positive focus score was accompanied by a significantly higher focus score (*P* < 0.001), relative area of CD45^+^ infiltrate (*P* < 0.001), number of CD3^+^ T-lymphocytes/mm^2^ (*P* < 0.001) and number of CD20^+^ B lymphocytes/mm^2^ (*P* = 0.018) in labial gland biopsies compared with their paired parotid gland biopsies. Labial salivary gland biopsies also more frequently exhibited CD21^+^ FDC networks (*P* = 0.004). While Bcl6^+^ GCs were identified as often in the two salivary gland types, the number of Bcl6^+^ GCs/mm^2^ was significantly higher in parotid gland biopsies (*P* = 0.016) (Table 3).

**Table 2.** Presence of histopathological key features in paired salivary gland sections of the total study population (non-SjD sicca and SjD patients, *n* = 99)

Parotid salivary gland	Labial salivary gland	
	Focus score ≥1	Focus score <1
Focus score ≥1	19 (19.2)	4 (4.0)
Focus score <1	16 (16.2)	60 (60.6)
	GC	No GC
GC	3 (3.0)	7 (7.1)
No GC	4 (4.0)	85 (85.9)
	IgA/IgG shift	No IgA/IgG shift
IgA/IgG shift	11 (11.1)	5 (5.1)
No IgA/IgG shift	11 (11.1)	72 (72.7)
	(Pre-)LEL	No (pre-)LEL
(Pre-)LEL	13 (13.1)	4 (4.0)
No (pre-)LEL	3 (3.0)	79 (79.8)

Data are reported as *n* (%). GC: germinal centre, LEL: lymphoepithelial lesion.

**Table 3.** Histopathological data of labial and parotid salivary gland biopsies in SjD and non-SjD sicca patients

	SjD and non-SjD sicca patients (*n* = 99)		
	Labial SG	Parotid SG	*P*-value
Surface area of salivary gland section^a^	11.1 (8.2–15.2)	9.6 (6.3–12.8)	0.021
Salivary gland section <4 mm^2^ (%)	1 (1)	7 (7)	0.07
Focus score^a^	0.5 (0.0–12.0)	0.0 (0.0–12.0)	<0.001
Infiltrated area (CD45^+^ cells) (%)	11.0 (7.0–19.2)	0.8 (0.3–6.2)	<0.001
FDC networks/mm^2^^a^	0.0 (0.0–0.6)	0.0 (0.0–1.2)	0.65
Presence of FDC networks, *n* (%)	31 (31.3)	17 (17.2)	0.004
GCs/mm^2^^a^	0.0 (0.0–0.2)	0.0 (0.0–0.5)	0.016
Presence of GCs, *n* (%)	7 (7.1)	10 (10.1)	0.72
CD3/CD20 segregation, *n* (%)^a^	27 (27.3)	22 (22.2)	0.30
IgA/IgG plasma-cell shift, *n* (%)^a^	28 (28.3)	20 (20.2)	0.08
LELs/mm^2^^a^	0.0 (0.0–0.4)	0.0 (0.0–1.0)	0.08
Presence of (pre-)LELs, *n* (%)	16 (16.2)	19 (19.2)	1.00
CD3^+^ cells/mm^2^	332 (200–564)	144 (74–330)	<0.001
CD20^+^ cells/mm^2^	90 (44–236)	24 (11–179)	0.018

Data are reported as median (IQR).

a Median (range) or *n* (%). Underlined values indicate *P*-values <0.05. SjD: Sjögren’s disease; SG: salivary gland; LEL: lymphoepithelial lesion; FDC: follicular dendritic cell; GC: germinal centre.

#### Subgroup diagnosed as SjD

**Figure 1.**
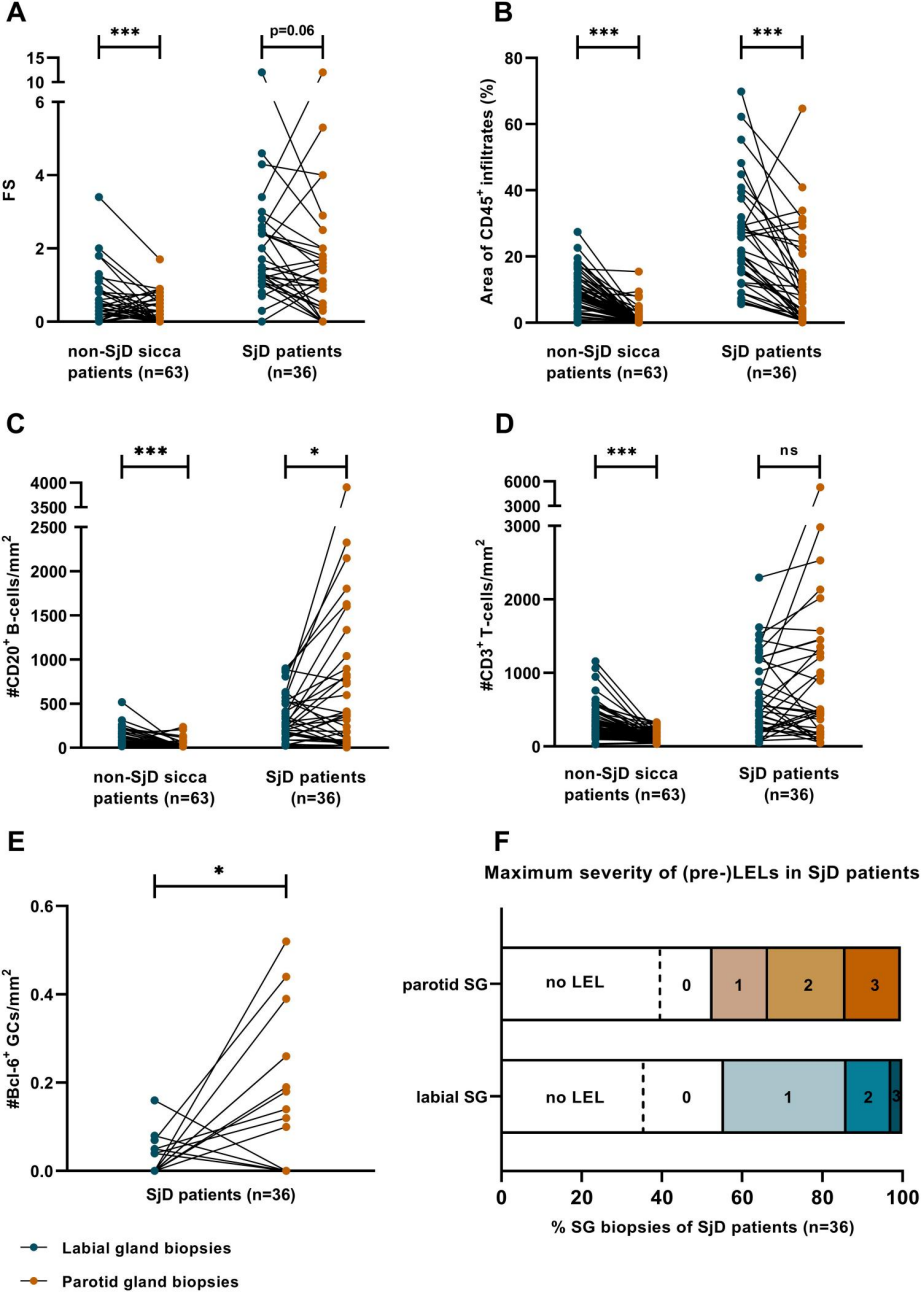
Histopathological comparison of labial and parotid salivary gland biopsies. Focus score (**A**), relative area of CD45^+^ infiltrates (**B**), CD20^+^ B lymphocytes (**C**) and CD3^+^ T cells (**D**) in SjD and non-SjD sicca patients and the number of Bcl6^+^ GCs per mm^2^ (**E**) and the maximum severity of LELs (**F**) in salivary gland sections of only SjD patients. LEL stage 0: pre-LEL, lymphocytic infiltration without ductal hyperplasia; LEL stage 1: lymphocytic infiltration with ductal hyperplasia affecting <50% of the epithelium; LEL stage 2: lymphocytic infiltration with ductal hyperplasia affecting 50–100%; LEL stage 3: lymphocytic ductal infiltration and fully circumferentially hyperplastic epithelium without lumen.^*^P < 0.05, ^***^P ≤ 0.001. SjD: Sjögren’s disease; LEL: lymphoepithelial lesion

Second, we compared the paired salivary gland biopsies of patients that were diagnosed as SjD based on the decision of the expert panel. Absolute agreement between labial and parotid glands was moderate to good, being 61% for the focus score, 69% for GCs, 58% for the IgA/IgG plasma-cell shift and 81% for (pre-)LELs (see Table 4). Higher focus scores (*P* = 0.06) and relative area of CD45^+^ infiltrates (*P* < 0.001) were observed in the labial compared with parotid glands, in line with the total study population (SjD and non-SjD sicca patients together) (Fig. 1A and B). Remarkedly, and in contrast to the total population, the number of CD20^+^ B lymphocytes/mm^2^ (*P* = 0.046) was lower in paired labial compared with parotid gland biopsies of SjD patients (Fig. 1C). Number of CD3^+^ T cells/mm^2^ (*P* = 0.29) (Fig. 1D) and the maximum organization grade of infiltrates per section (*P* = 0.65) were comparable between paired labial and parotid gland biopsies of SjD patients. Although the number of biopsies which harboured GCs or (pre-)LELs did not differ between the two types of glands, the number of GCs/mm^2^ (*P* = 0.016) and severity of LELs were significantly lower in labial gland biopsies (*P* = 0.026) (Fig. 1E and F). Almost all salivary gland biopsies (5/7 labial gland biopsies, 10/10 parotid gland biopsies) which exhibited GCs as detected by Bcl6, also revealed clusters of five or more AID^+^ cells and vice versa. Of note, in 14/21 labial and 5/15 parotid salivary gland biopsies with CD21^+^ FDC networks, no Bcl6^+^ and/or AID^+^ clusters were observed (Supplementary Fig. S3, available at *Rheumatology* online).

**Table 4.** Histopathological key features in paired salivary gland sections of SjD patients (*n* = 36)

Parotid salivary gland	Labial salivary gland	
	Focus score ≥1	Focus score <1
Focus score ≥1	19 (52.8)	4 (11.1)
Focus score <1	10 (27.8)	3 (8.3)
	GC	No GC
GC	3 (8.3)	7 (19.4)
No GC	4 (11.1)	22 (61.1)
	IgA/IgG shift	No IgA/IgG shift
IgA/IgG shift	11 (30.6)	4 (11.1)
No IgA/IgG shift	11 (30.6)	10 (27.8)
	(Pre-)LEL	No (pre-)LEL
(Pre-)LEL	13 (36.1)	4 (11.1)
No (pre-)LEL	3 (8.3)	16 (44.4)

Data are reported as *n* (%). SjD: Sjögren’s disease; GC: germinal centre, LEL: lymphoepithelial lesion.

#### Subgroup diagnosed as non-SjD

Third, we compared the paired biopsies non-SjD sicca patients. A significantly higher focus score (*P* < 0.001), relative area of CD45^+^ infiltrates (*P* < 0.001), number of CD3^+^ T lymphocytes (*P* < 0.001) and CD20^+^ B lymphocytes (*P* < 0.001) was seen in labial salivary gland biopsies compared with parotid gland biopsies (Fig. 1). The grade of organization of infiltrates was comparable between the two paired salivary gland types in non-SjD sicca patients. GCs, IgA/IgG plasma-cell shift or (pre-)LELs were virtually absent in non-SjD sicca patients, except for two parotid gland biopsies in which an IgA/IgG shift (*n* = 1) or a pre-LEL (*n* = 1) were observed.

### Correlation analysis between paired labial and parotid gland biopsies

**Figure 2.**
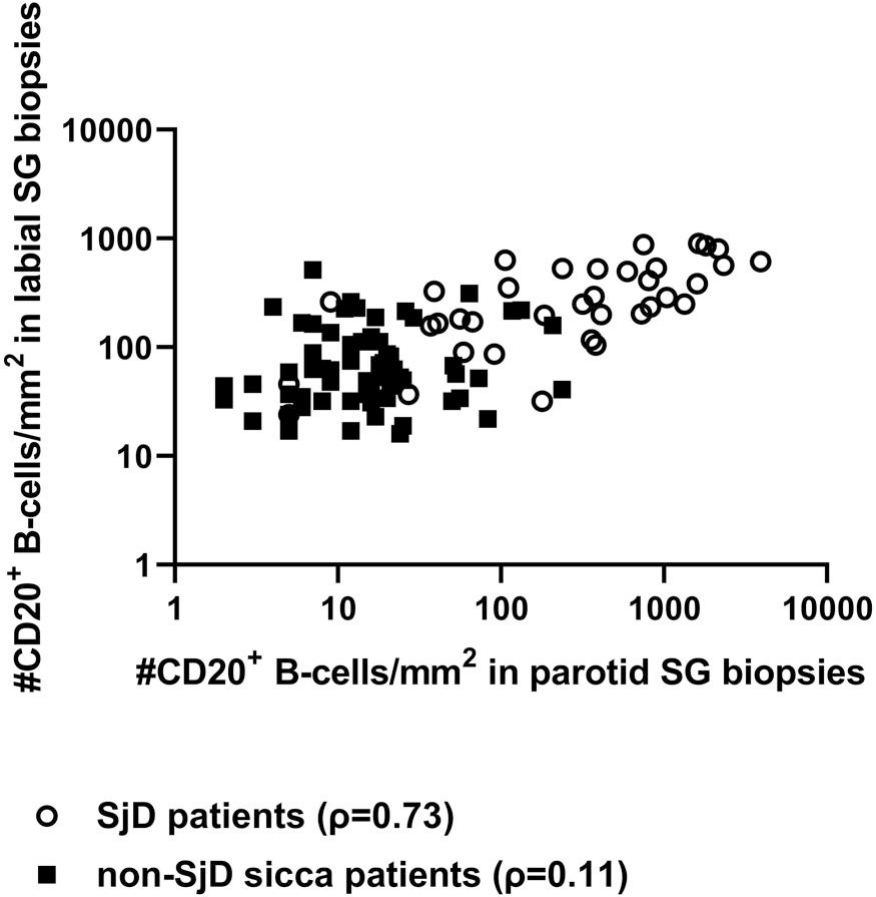
Association between the number of CD20^+^ B lymphocytes in paired labial and parotid salivary gland biopsies of non-SjD sicca patients and SjD patients. SjD: Sjögren’s disease

In order to assess to what extent the various histopathological features are present in the two types of glands, associations of the features between paired labial and parotid gland biopsies were determined. Correlation of all analysed parameters varied from fair to good in the total study population. When taking only SjD patients into account, correlations were moderate to good for most features, except for fair correlations for the number of FDC networks/mm^2^ and GCs/mm^2^. Interestingly, for the SjD patients, the number of CD20^+^ B lymphocytes showed a good correlation between the gland types, while for the non-SjD sicca patients a poor correlation was observed (ρ = 0.73 *vs* ρ = 0.11). This was mainly due to a discrepancy in B-lymphocyte numbers between gland types in non-SjD sicca patients (Fig. 2). Similar correlation coefficients were found for the total study population and SjD patients for focus score and area of CD45^+^ infiltrate (Supplementary Table S3, available at *Rheumatology* online).

## Discussion

In a diagnostic cohort of sicca patients from daily clinical practice, paired labial and parotid salivary gland histopathology appeared comparable in SjD patients. Importantly, absolute agreement of labial glands and parotid glands in terms of the SjD-related features, focus score, presence of GCs, plasma cell isotype switch and LELs was high, and correlation of most features between the two salivary gland types was generally moderate to good. However, important histopathological differences were also observed.

More and larger lymphocytic infiltrates were seen in labial glands compared with parotid glands, not only in SjD patients, but also in non-SjD sicca patients. This was reflected by a higher focus score and amount of infiltrate, as well as numbers of T and B lymphocytes in the labial glands. The higher number of infiltrating lymphoid cells in labial glands of non-SjD sicca patients argues that lymphocytic infiltrates are frequently present in these glands irrespective of the presence of SjD. The lymphocytic infiltrates in labial glands may develop in SjD patients on top of non-autoimmune related infiltrates, resulting in a higher amount of infiltrate in labial glands also in SjD patients. Reasons for the presence of non-autoimmune-related infiltrates in labial glands most clearly seen in non-SjD sicca patients are unknown, but possibilities are gland dysfunction, worse oral health, infections or habitual lip biting [21]. Also dysbiosis in the buccal mucosa microbiome observed in non-SjD sicca patients may contribute to inflammation in the labial salivary glands [22]. Furthermore, labial salivary glands are more easily accessible to microbes in comparison with the parotid gland, primarily due to anatomical differences such as size and length of the excretory ducts. As a consequence of the presence of non-autoimmune related infiltrates in labial glands, patients are potentially misclassified as SjD patients when solely the labial gland focus score is used as a histopathological diagnostic criterion. Specificity of the labial gland biopsy for SjD is increased when taking into account not only the focus score, but also other characteristic histopathological features of SjD, i.e. presence of GCs, plasma cell isotype switch, and (pre-)LELs (van Ginkel *et al.* manuscript in press) [18].

In this study, we unequivocally showed the presence of bona fide GCs in both type of glands by staining for Bcl6 and AID. The small discrepancy seen in the number of biopsies with GCs in labial gland biopsies based on either Bcl6 or AID staining can be explained by the fact that the sections stained for these two markers were not adjacent sections.

In this study, we observed that while the number of biopsies with a GC did not significantly differ between paired salivary gland biopsies, more GCs/mm^2^ were found in parotid gland biopsies. The higher number of GCs in parotid gland biopsies may indicate that there is a more active humoral immune response in these glands, compared with the labial glands. In addition, a higher absolute B-lymphocyte count was observed in parotid gland biopsies compared with paired labial gland biopsies in SjD patients.

A higher number of B lymphocytes may have implications for other histopathological features. B lymphocytes can invade the epithelium of the striated ducts, which probably drives the formation of LELs [16]. Haacke *et al.* showed that the majority of the intraepithelial B lymphocytes in SjD patients express the inhibitory Fc-receptor like 4 (FcRL4) protein, which is also abundantly expressed by MALT lymphoma B lymphocytes in SjD [23]. There are more FcRL4^+^ B lymphocytes in parotid glands, compared with labial glands, and these cells actively clonally expand within the epithelium [24, 25]. We hypothesized that during this expansion derailment of intraepithelial FcRL4^+^ B lymphocytes may result in MALT lymphoma formation, in particular in the parotid gland environment [25]. Taken together, these findings indicate that within the parotid salivary glands there seems to be more pronounced B-lymphocyte activity which is at least partly responsible for the higher number of GCs, more severe LELs and MALT lymphoma development.

The reason for differences in B-lymphocyte numbers and activity between the two types of glands in SjD remains to be elucidated. It is possible that higher levels of certain pro-inflammatory cytokines [e.g. IFN-γ, IL-27, B-cell-activating factor of the TNF family (BAFF) and a proliferation-inducing ligand (APRIL)] result in more attraction and/or activation of B lymphocytes in parotid glands [25]. However, transcriptomic analysis of paired parotid and labial salivary gland biopsies of SjD patients showed a high degree of overlap in immune pathway activity between the two salivary gland types [26].

In conclusion, both labial and parotid gland biopsies have similar histopathological key features and both types of salivary glands can be used for diagnosis and classification of SjD. However, systematic analysis of paired salivary gland biopsies also revealed important differences between these two glands. Labial salivary glands seem to exhibit more non-SjD-related inflammation which can obscure diagnosis and classification. In SjD, parotid salivary glands reveal more evident histopathological signs of B-lymphocyte hyperactivity. The results of this study offer novel insights into the pathophysiology of SjD and can be incorporated into guidelines for the histopathological analysis of salivary gland biopsies.

## Supplementary Material

keae154_Supplementary_Data

## Data Availability

Data available on request from the authors.
