# Supplementary material for: Histopathological comparison of Sjögren-related features between paired labial and parotid salivary gland biopsies of sicca patients
Source: Rheumatology (Oxford). 2024 Mar 27;63(10):2670–7. doi: 10.1093/rheumatology/keae154 (PMC11443036; doi:10.1093/rheumatology/keae154)
Supplement: keae154_Supplementary_Data [file keae154_supplementary_data.docx]

**SUPPLEMENTARY MATERIAL**

**Supplementary Table S1. Primary antibodies and staining method used for immunohistochemistry.**

| Antigen | Clone | Host | Source | Staining method |
| --- | --- | --- | --- | --- |
| AID-2  CD3  CD20  CD21  CD45  Bcl6  IgA/IgG  hmwCK | mAID-2  2GV6  L-26  2G9  2B11+PD7/26  GI19E/A8  Polyclonal  34βE12 | Rat  Rabbit  Mouse  Mouse  Mouse  Mouse  Rabbit  Mouse | Thermo Fisher Scientific  Ventana Roche  Ventana Roche  Cell Marque Corporation  Ventana Roche  Ventana Roche  Ventana Roche  Ventana Roche | Manually  Manually  Manually  Automated  Automated  Automated  Automated  Manually |

**
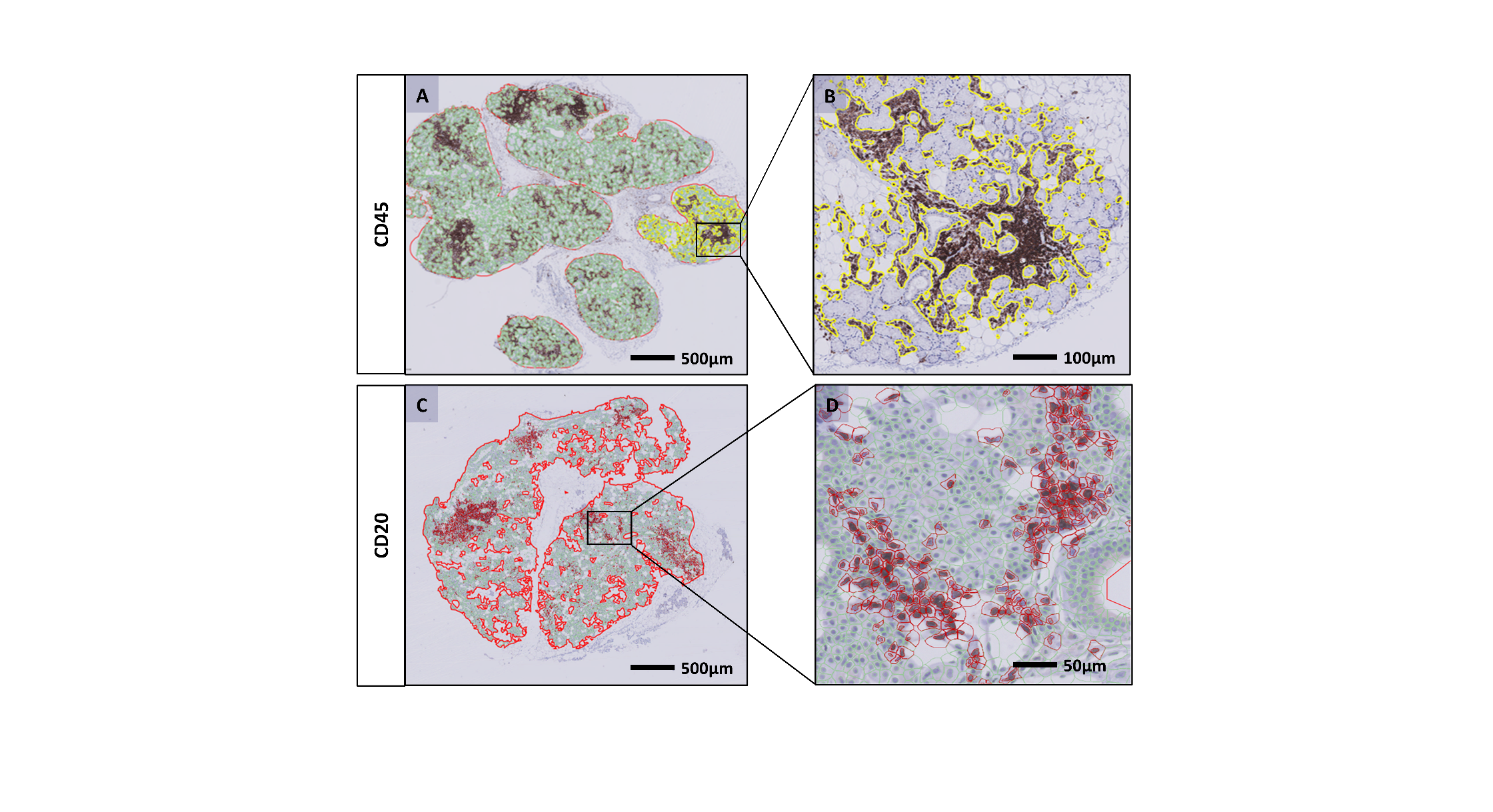
**

**Supplementary Figure S1. Quantitative digital image analyses (DIA) of salivary gland sections stained for CD45+ lymphocytic infiltrates and CD20+ B-lymphocytes using QuPath v0.1.2.** For each section, the total area of parenchyma was evaluated by defining regions of interest using the Simple Tissue Detection application, excluding extra- and intra-parenchymal areas with adipose tissue. Hereafter, extra-parenchymal fibrotic and atrophic areas were manually excluded resulting in red encircled areas. Relative area of infiltrates within a labial salivary gland section was assessed by staining for CD45. The total area of ‘positive staining’ (yellow encircled areas) was calculated and expressed as a percentage per mm2 glandular tissue (A-B). Absolute and relative B-cell counts within a parotid salivary gland section were analysed by staining for CD20. DAB+ cells (red encircled in D) were selected, quantified and expressed as number per mm2. The method illustrated in C-D was used for sections stained for CD3 and CD20.

**
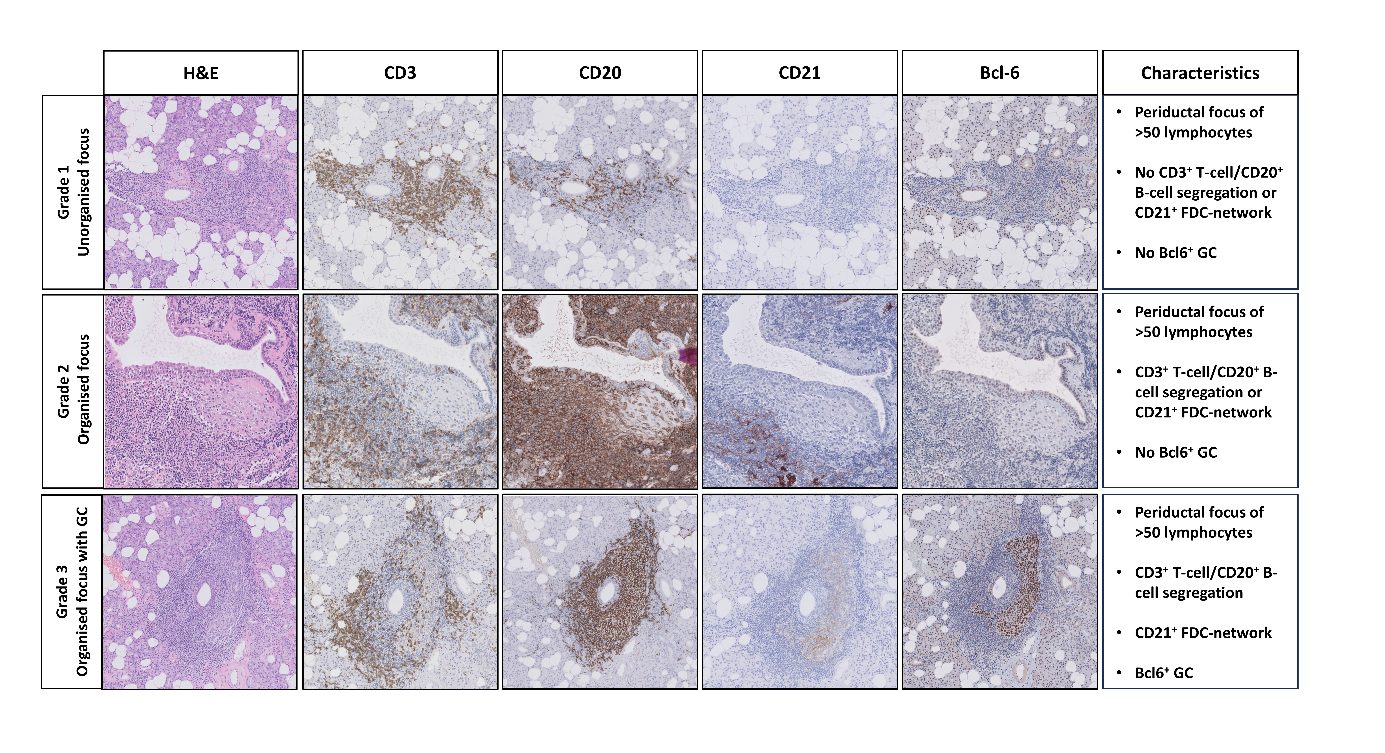
**

**Supplementary Figure S2. Grading of lymphoid organisation in salivary glands of SjD patients.** Grade 1: lymphocytic foci were present, but without expressing a clear T/B-cell segregation in the CD3 and CD20 stainings, without presence of a FDC-network (CD21^+^) and without presence of a GC (Bcl6^+^). Grade 2: either a T/B cell segregation within a focus and/or an FDC-network was present, but without presence of a GC. Grade 3: grade 2 features accompanied by the presence of a GC. Sections derived from parotid salivary gland biopsy.

**
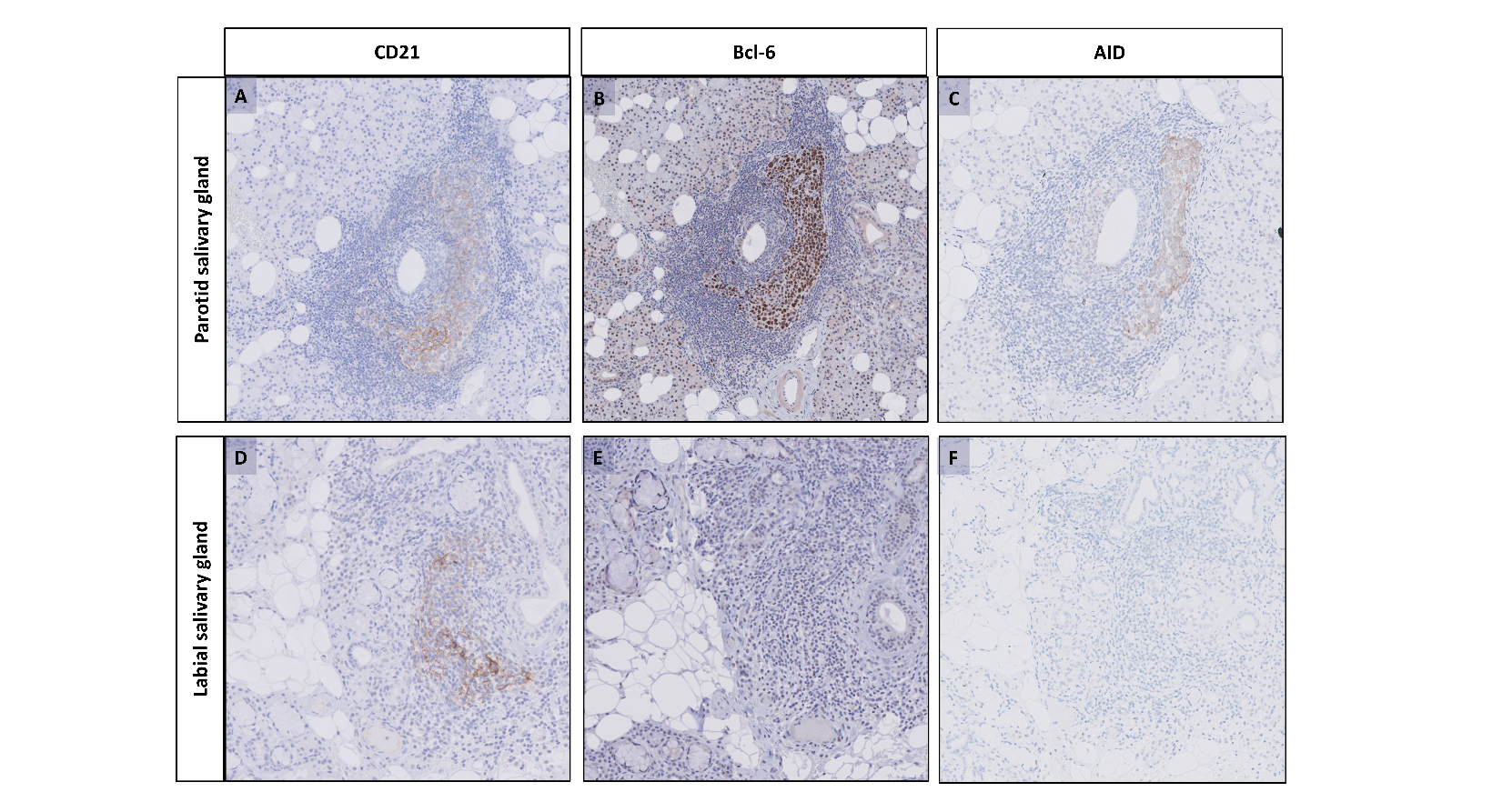
**

**Supplementary Figure S3. Salivary gland biopsies of two SjD patients stained for CD21, Bcl-6 and AID.** In the first row (A-C) a parotid gland biopsy with a periductal focus with presence of CD21^+^ FDC-network with Bcl6^+^ GC and expression of AID. Whereas in the second row (D-F) a labial gland biopsy with a CD21^+^ FDC-network is present without Bcl6 or AID express.

**Supplementary Table S2. Detailed characteristics of sicca patients with discrepancy between expert opinion and ACR-EULAR classification criteria.**

| Patient number | Expert opinion | Fulfilment ACR-EULAR classification criteria | Total ACR-EULAR points | FS Labial SG | FS Parotid SG | Anti-SSA/Ro titer | Anti-SSB/La titer | IgG g/L | RF titer IU/ml | OSS | Schirmer’s test mm/min (lowest) | UWS ml/min | SWS ml/min | Total ESSDAI score | Total ESSPRI score |
| --- | --- | --- | --- | --- | --- | --- | --- | --- | --- | --- | --- | --- | --- | --- | --- |
| 1 | SjD | No | 3 | 0,7 | **1,5** | 0 | 0 | 8.6 | 1.2 | 0 | 6 | 0.4 | 1.1 | 18 | 8 |
| 2 | SjD | No | 3 | **1,4** | **1,7** | 0 | 0 | 10.7 | 0 | 0 | 29 | 0.2 | 0.9 | 2 | 4 |
| 3 | Non-SjD Sicca | Yes | 4 | 0 | 0 | **164** | 0 | 9.9 | 1.3 | **8** | 18 | 0.2 | 1.2 | 5 | 7 |
| 4 | Non-SjD Sicca | Yes | 4 | **1,2** | 0,9 | 0 | 0 | 14.4 | 1.3 | 1 | **2** | 0.2 | 0.6 | 3 | 8.3 |
| 5 | Non-SjD Sicca | Yes | 4 | 0,4 | 0,6 | **240** | 0 | 12.3 | 0 | 2 | **5** | 0.2 | 0.2 | 7 | 6 |
| 6 | Non-SjD Sicca | Yes | 4 | **1,3** | 0 | 0 | 0 | 9.6 | 2.1 | 1 | **3** | 0.3 | 1.0 | 1 | 7 |
| 7 | Non-SjD Sicca | Yes | 4 | **1,8** | 0 | 0 | 0 | 18.2 | 6 | 0 | 6 | **0.0** | 0.0 | 5 | 7 |
| 8 | Non-SjD Sicca | Yes | 4 | **1,8** | 0,5 | 0 | 0 | 11.5 | 0 | 3 | **4** | 0.4 | 0.5 | 0 | 8 |
| 9 | Non-SjD Sicca | Yes | 5 | **2,0** | 0,6 | 0 | 0 | 10.6 | 0.9 | **7** | **5** | 0.3 | 0.9 | 0 | 7 |
| 10 | Non-SjD Sicca | Yes | 5 | 0 | 0 | **233** | 0 | 11.5 | 1.4 | 0 | **4** | **0.01** | 0.3 | 2 | 9 |
| 11 | Non-SjD Sicca | Yes | 6 | **3,4** | **1,7** | **17** | 0 | 8.4 | 0.6 | 0 | 35 | 0.3 | 1.7 | 6 | 9 |

FS, focus score; SSA, Sjögren’s syndrome antigen A; OSS, Oscular staining score; UWS, unstimulated whole saliva; SWS, stimulated whole saliva L, Labial salivary gland. Scores that are positive according to the ACR-EULAR criteria are shown in bold.

|  | **Total study population (n=99)** | **SjD patients (n=36)** | **Non-SjD sicca patients (n=63)** |
| --- | --- | --- | --- |
| **FS** | 0.60* | 0.62* | 0.31* |
| **%CD45** | 0.64* | 0.65* | 0.37* |
| **LELs/mm^2^** | 0.73* | 0.56* | n/a |
| **CD3^+^ T-cell/mm^2^** | 0.34* | 0.45* | 0.24 |
| **CD20^+^ B-lymphocytes/mm^2^** | 0.52* | 0.73* | 0.11 |
| **Bcl6^+^ GCs/mm^2^** | 0.39* | 0.27 | n/a |
| **CD21^+^ FDCs/mm^2^** | 0.47* | 0.25 | 0.44* |

**Supplementary Table S3. Correlation coefficients of histopathological features in labial and parotid gland biopsies of the total study population and stratified for SjD patients and non-SjD sicca patients.**


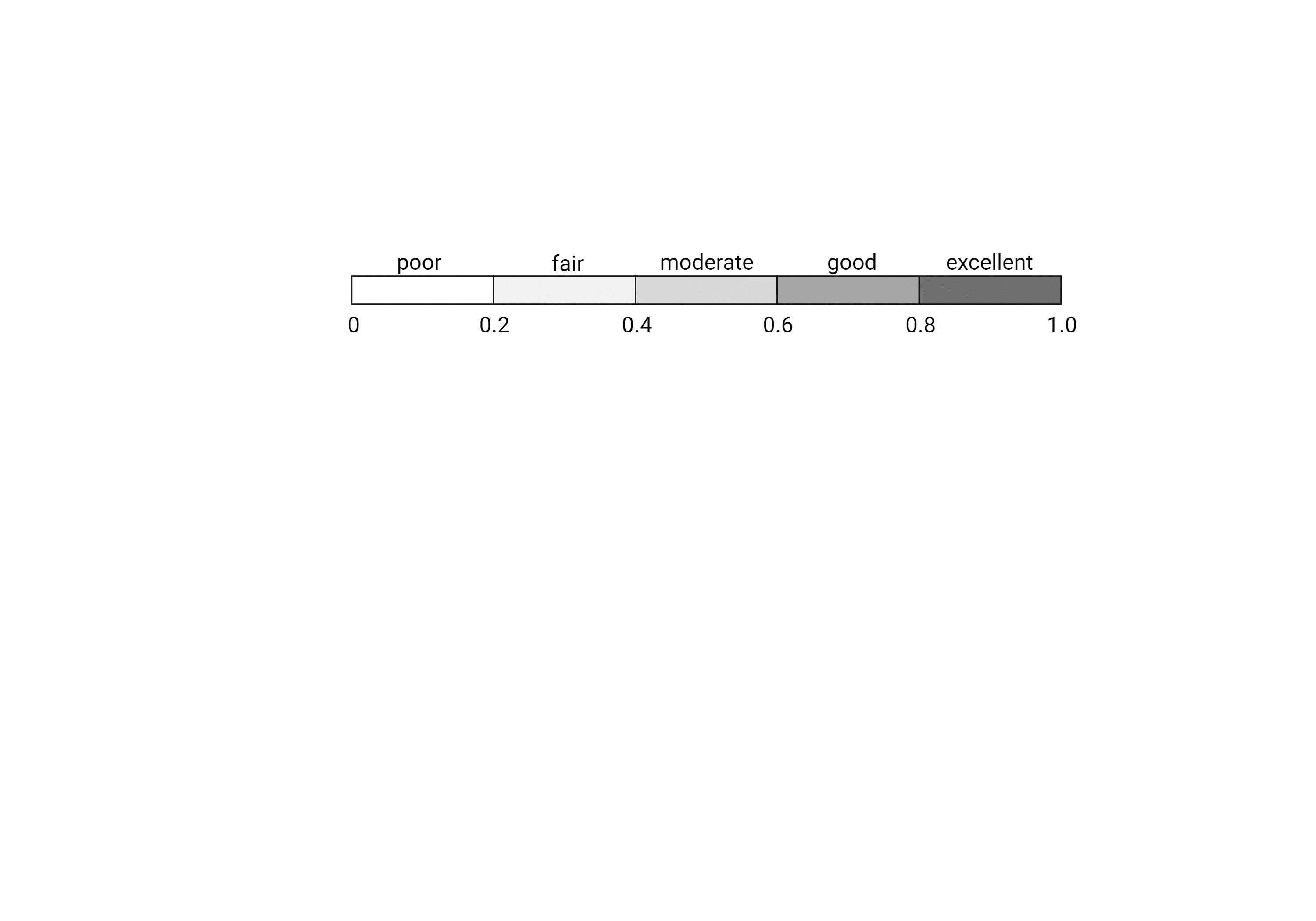


*P<0.05
